# AU-Rich Long 3′ Untranslated Region Regulates Gene Expression in Bacteria

**DOI:** 10.3389/fmicb.2018.03080

**Published:** 2018-12-12

**Authors:** Ju-Ping Zhao, Hui Zhu, Xiao-Peng Guo, Yi-Cheng Sun

**Affiliations:** MOH Key Laboratory of Systems Biology of Pathogens, Institute of Pathogen Biology, Chinese Academy of Medical Sciences and Peking Union Medical College, Beijing, China

**Keywords:** 3′ UTR, AU-rich region, post-transcriptional regulation, mRNA stability, *Yersinia pestis*

## Abstract

3′ untranslated regions (3′ UTRs) and particularly long 3′ UTRs have been shown to act as a new class of post-transcriptional regulatory element. We previously reported that *hmsT* mRNA stability is negatively regulated by the 3′ UTR of *hmsT* in *Yersinia pestis*. To investigate more general effects of 3′ UTRs in *Y. pestis*, we selected 15 genes potentially possessing long 3′ UTRs with different AU content and constructed their 3′ UTR deletion mutants. Deletion of AU-rich 3′ UTRs increased mRNA levels, whereas deletion of 3′ UTRs with normal AU content resulted in slight or no changes in the mRNA level. In addition, we found that PNPase was important for 3′ UTR-mediated mRNA decay when the transcriptional terminator was Rho-dependent. Finally, we showed that ribosomes promote mRNA stability when bound to a 3′ UTR. Our findings suggest that functional 3′ UTRs might be broadly distributed in bacteria and their novel regulatory mechanisms require further investigation.

## Introduction

To adapt to changes in the environment, an intricate regulatory network that accurately modulates gene expression has evolved in bacteria. Gene expression in bacteria is primarily regulated at the transcriptional level. However, unlike transcriptional regulation, post-transcriptional regulation of gene expression allows bacteria to adjust rapidly to the changing environment. One way in which post-transcriptional regulation is achieved is via changes in mRNA stability (Laalami et al., 2014).

5′ and 3′ untranslated regions (UTRs) contain many elements that assist in the regulation of gene expression (Pesole et al., 2001). 5′ UTR mediated gene regulation has been extensively studied in bacteria (Oliva et al., 2015). The 5′ UTR reportedly folds into a specific secondary structure, such as an RNA thermometer or riboswitch (Henkin, 2008; Breaker, 2011; Kortmann and Narberhaus, 2012; Serganov and Patel, 2012; Krajewski and Narberhaus, 2014). In addition to secondary structures, ribosome binding to the Shine-Dalgarno sequence increases the stability of the downstream mRNA (Agaisse and Lereclus, 1996; Sharp and Bechhofer, 2003; Daou-Chabo et al., 2009). Traditionally, bacterial 3′ UTRs were thought to comprise mainly transcriptional terminators. However, transcriptional terminators rarely exceed a size of 40–50 nucleotides; thus 3′ UTRs of greater length (>100 nt) have been hypothesized to contain other regulatory elements (Ruiz de los Mozos et al., 2013). Recent work has shown that 3′ UTRs are involved in regulating gene expression in bacteria (Ren et al., 2017). In particular, 3′ UTRs regulate the decay rate and translational initiation of mRNAs(Selinger et al., 2003; Maeda and Wachi, 2012; Lopez-Garrido et al., 2014; Liu et al., 2016; Zhu et al., 2016). Regulatory small RNAs (sRNAs) target 3′ UTRs, which in turn regulates the expression of the UTR-containing mRNAs (Opdyke et al., 2004; Silvaggi et al., 2005). In addition, 3′ UTRs provide a rich reservoir of sRNAs for the targeted regulation of gene expression (Chao et al., 2012; Gossringer and Hartmann, 2012; Kim et al., 2014; Tree et al., 2014; Miyakoshi et al., 2015; Chao and Vogel, 2016; Peng et al., 2016). The RNA chaperone Hfq binds to the 3′ ends of mRNAs (Holmqvist et al., 2016) and protects RNA from 3′ to 5′ exonuclease activity (Hajnsdorf and Regnier, 2000; Le Derout et al., 2003).

Several 3′ UTRs are reportedly attacked by ribonucleases to initiate mRNA degradation. One early example is the 3′ UTR of *aceA* in *Corynebacterium glutamicum*, which contains an AU-rich region cleaved by RNase E/G (Maeda and Wachi, 2012). Although the *aceA* 3′ UTR is only 63 nt in length, other 3′ UTRs involved in mRNA decay are much longer. For example, the 3′ UTR of *hilD* mRNA is 310 nt and is degraded by RNase E and polynucleotide phosphorylase (PNPase) in *Salmonella enterica* (Lopez-Garrido et al., 2014). Another example is the 3′ UTR of *hmsT*, which is 283 nt in length and cleaved by PNPase in *Yersinia pestis* (Zhu et al., 2016). The 3′ UTRs of *hilD* and *aceA* possess a specific AU-rich regulatory region (Maeda and Wachi, 2012; Lopez-Garrido et al., 2014). Although the *hmsT* 3′ UTR does not contain a specific AU-rich regulatory element, it is rich in AU nucleotides (70%). Thus, AU-rich 3′ UTRs appear to be susceptible to RNase attack, which in turn initiates mRNA decay.

*Y. pestis* causes plague and has two hosts: mammals and fleas. *Y. pestis* undergoes significant change when it is transmitted from the flea vector to the mammalian host. Recently, we found that the 3′ UTR increases *hmsT* mRNA decay at the body temperature of the mammalian host but not at the body temperature of the flea vector (room temperature) (Zhu et al., 2016). In the present study, to investigate the functions of 3′ UTRs in *Y. pestis* in greater detail, we analyzed 15 *Y. pestis* genes potentially possessing long 3′ UTRs but with differences in AU-content. The results showed that AU-rich 3′ UTRs can negatively regulate the expression of their own genes. Our findings suggest that functional 3′ UTRs are widespread in bacteria and warrant further investigations to uncover their regulatory mechanisms.

## Results

### Investigation of the General Effects of Long 3′ UTRs on Gene Expression in *Y. pestis*

**FIGURE 1:**
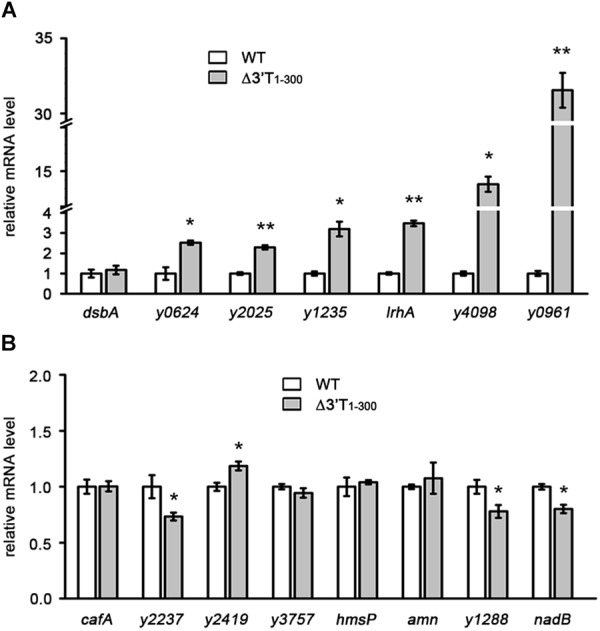
Characterization of the general effects of 3′ UTRs on mRNA expression in *Yersinia pestis*. **(A,B)** qRT-PCR analysis of transcripts of genes containing AT-rich **(A)** or normal **(B)** 3′ IGRs in the wild type (WT) and nucleotides 1–300 of the 3′ terminus (3′ T_1-300_) mutant strains (Δ3′T_1-300_), in which the 3′ T_1-300_ is disrupted by insertion of a kanamycin (kan) cassette. The *Y. pestis* WT strain was used as a control, and the fold change in relative mRNA level for each strain was compared with that of the WT (set as 1). Student’s *t*-test was used to calculate *p*-values. ^∗^*p* < 0.05, ^∗∗^*p* < 0.001. Means ± standard deviation (SD) from three independent experiments are shown.

Long 3′ UTRs play an important role in mRNA turnover (Selinger et al., 2003; Maeda and Wachi, 2012; Liu et al., 2016; Zhu et al., 2016). More interestingly, these long 3′ UTRs are usually rich in AU nucleotides (Maeda and Wachi, 2012; Lopez-Garrido et al., 2014; Zhu et al., 2016), indicating that AU-rich elements might be important for 3′ UTR-mediated mRNA decay. To investigate the role of AU-rich elements and the general effects of 3′ UTRs on mRNA turnover, 15 genes with long 3′ intergenic regions (IGRs > 250 bp) were randomly selected from the *Y. pestis* genome (Table 1). Seven genes had high (>68%) and eight genes had normal (45–60%) AT content in their 250 bp-3′ IGRs compared with the 52% AT content of the *Y. pestis* chromosome (Parkhill et al., 2001; Deng et al., 2002) (Table 1). Nucleotides 1–300 of the 3′ terminus (3′ T) of the 15 selected genes were deleted and replaced with a kanamycin (kan) cassette, and their mRNA levels were compared between wild-type (WT) and 3′ T_1-300_ deletion mutants using quantitative real-time PCR (qRT-PCR). As shown in Figure 1, for six of seven genes with high AT content (>68%) in their 3′ T regions, mRNA levels were significantly increased when the 3′ T region (potential 3′ UTR) was deleted. By contrast, for genes with normal AT content, only one gene (*y2419*, AT content = 49% in its 3′ T region) displayed a slightly increased mRNA expression when its 3′ T region (potential 3′ UTR) was deleted, whereas three genes (*y2237*, *y1288*, and *nadB* with AT content = 48%, 52%, and 46% in their 3′ T region, respectively) displayed slightly decreased mRNA expression.

**Table 1:** Analysis of potential 3′ UTRs of 17 genes with long 3′ IGRs in *Y. pestis*.

Gene name	IGR length (bp)	AT% in 3′ terminus (1–250 bp)	TTS (bp)^∗^	AT% in potential 3′ UTR
*hmsT*	540	73%	283^∗^	70%
*dsbA*	491	70%	48^∗∗^	69%
*y0624*	314	72%	Unknown	–
*y2025*	837	68%	63^∗∗^	59%
*y1235*	371	70%	250	70%
*lrhA*	677	70%	60^∗∗^	72%
*y4098*	349	69%	284	68%
*y0961*	459	70%	Unknown	–
*cafA*	825	45%	Unknown	–
*y2237*	592	48%	223^∗∗^	45%
*y2419*	661	49%	Unknown	–
*y3757*	540	57%	Unknown	–
*hmsP*	639	60%	Unknown	–
*amn*	424	55%	Unknown	–
*y1288*	282	52%	Unknown	–
*nadB*	282	46%	243^∗∗^	46%


*IGR, intergenic region; TTS, transcriptional termination site. ^∗^TTS of *hmsT* mRNA was determined by the 3′ RACE analysis as described in our previous study (Zhu et al., 2016). ^∗∗^Predicted Rho-independent TTS was determined based on the ARNold program. “Unknown” indicates the TTS is unknown.*

**FIGURE 2:**
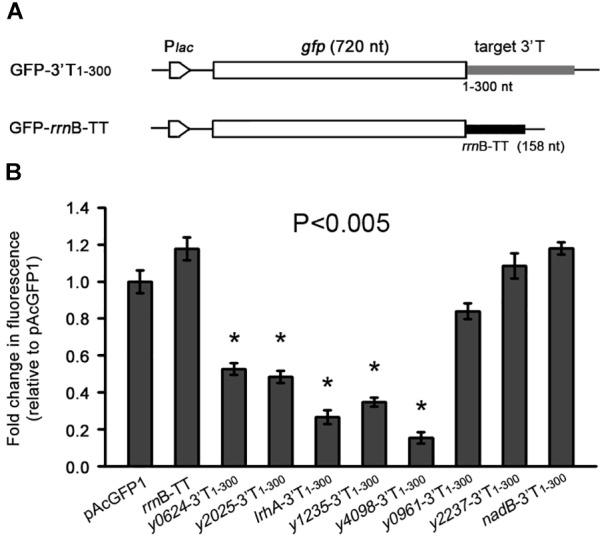
The effects of 3′ UTRs on expression of a heterologous green fluorescent (GFP) reporter. **(A)** Schematic representation of GFP reporter with different 3′ UTRs and an *rrnB* transcriptional terminator. **(B)** Relative GFP fluorescence of *E. coli* cells expressing GFP with different 3′ UTRs (GFP-3′T_1-300_) and the *rrnB* terminator, and without an additional 3′ UTR (pAcGFP1). Relative fluorescence intensities were normalized against the cell density (OD_600_ value) of the bacterial culture, and the fold change in fluorescence was compared with that of the vector control (set as 1). Student’s *t*-tests were used to calculate *p*-values. ^∗^*p* < 0.01. Means ± SD from three independent experiments are indicated.

To determine the role of these 3′ UTRs, we investigated their influence on the expression of a heterologous green fluorescent protein (*gfp)* gene. The 3′ T_1-300_ fragments of eight genes (six AT-rich and two normal) were cloned immediately into the downstream region of the *gfp* gene and the constructs were transformed into *Escherichia coli* cells, along with the vector control pAcGFP1 and a 3′ terminal control *rrn*B-TT (Figure 2A). As shown in Figure 2B, five 3′ terminal regions with high AT content caused a significant decrease in GFP fluorescence compared with the two controls (pAcGFP1 and *rrn*B-TT), whereas the *y2237* and *nadB* 3′ terminal regions, which have moderate AT content, did not significantly affect *gfp* expression. Taken together, the results indicate that five out of seven selected AU-rich 3′ UTRs acted as independent units for the regulation of heterogonous gene expression. These results imply that the effects of 3′ UTRs on mRNA turnover might be broadly distributed in *Y. pestis*, and long AU-rich 3′ UTRs may be intimately involved in mRNA turnover.

### The 3′ UTRs of *y1235* and *y4098* Negatively Regulate mRNA Stability

**FIGURE 3:**
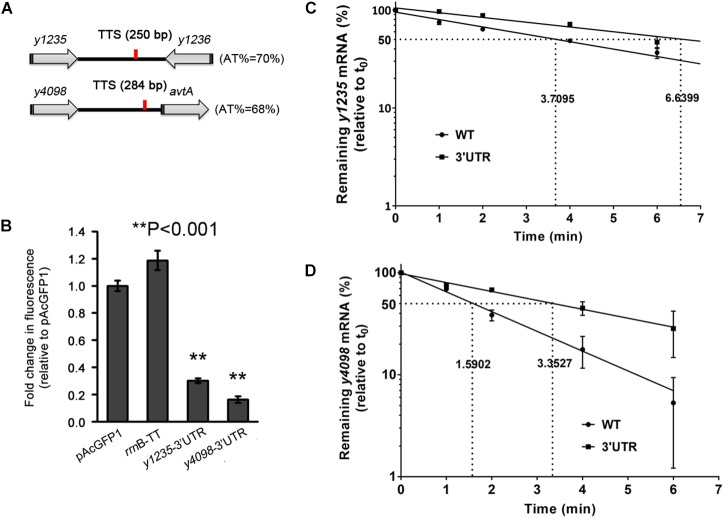
The 3′ UTRs of *y1235* and *y4098* negatively regulate mRNA stability. **(A)** Schematic representation of *y1235*, *y4098*, and their transcriptional terminator (TTS, red line). **(B)** Relative GFP fluorescence of *E. coli* cells expressing GFP with the 3′ UTR of *y1235* or *y4098*, or the *rrnB* terminator, or without an additional 3′ UTR (pAcGFP1). Relative fluorescence intensities were normalized against the OD_600_ value of the bacterial culture, and the fold change in fluorescence was compared with that of the vector control (set as 1). Student’s *t*-tests were used to calculate *p*-values. ^∗∗^*p* < 0.001. Means ± SD from three independent experiments are indicated. **(C,D)** The half-life of *y1235* and *y4098* mRNAs in WT and Δ3′ UTR mutant strains. Cells were grown at 26°C to an OD_600_ of 0.8, and rifampicin was added at time 0 to block RNA transcription. Samples were removed at time 0, 1 min, 2 min, 4 min, and 6 min. The percentage of residual mRNA at each time-point was measured by qRT-PCR and compared with that at time 0. Dashed lines indicate the time at which half of the detected mRNA remained. mRNA half-life was determined using linear regression analysis and is shown on the dashed lines. Error bars indicate SD of the mean.

To explore further the regulatory functions of the 3′ UTRs, those of *y1235* and *y4098*, which significantly affected *gfp* expression (Figure 2B), were selected for further study. Sequence analysis using the ARNold program (Naville et al., 2011) revealed that no Rho-independent transcriptional terminator (intrinsic terminator) was present downstream of *y1235* or *y4098*. 3′ rapid amplification of cDNA ends (RACE) data showed that the transcriptional termination sites (TTS) of *y1235* and *y4098* mRNAs are located 250- and 284-nt downstream of the stop codon, respectively (Figure 3A and Supplementary Figure S1). To further verify the role of these two 3′ UTRs, 250 and 284 bp fragments corresponding to the 3′ UTRs of *y1235* and *y4098* were cloned downstream of the *gfp* coding sequence, respectively. GFP reporter analysis showed that these constructs performed a similar regulatory role compared with their 3′ T_1-300_ regions (Figures 2B, 3B), further suggesting that the 3′ UTRs of *y1235* and *y4098* are involved in regulation of mRNA decay.

To determine whether the 3′ UTR affects the expression of *y1235* and *y4098* at the post-transcriptional level, the half-lives of *y1235* and *y4098* mRNAs were measured by qRT-PCR in WT and 3′ UTR deletion mutant *Y. pestis* strains. The *y1235* transcript half-life in the WT strain was 3.71 min, while the *y1235* transcript half-life in the *y1235* 3′ UTR mutant was 6.64 min (Figure 3C). Deletion of the 3′ UTR of the *y4098* transcript increased its half-life from 1.59 min to 3.35 min (Figure 3D). These results suggest that *y1235* and *y4098* 3′ UTRs regulate mRNA decay at the post-transcriptional level.

### Examination of the Role of Hfq in 3′ UTR-Mediated *y1235* and *y4098* mRNA Decay

**FIGURE 4:**
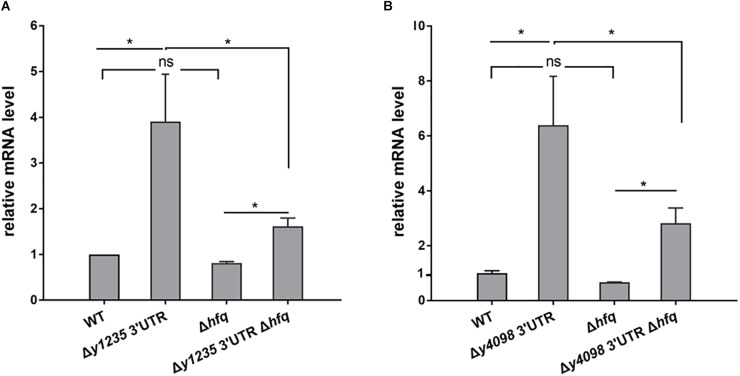
Examination of the roles of Hfq in 3′ UTR-mediated decay of *y1235* and *y4098* mRNA. Relative amounts of *y1235* **(A)** and *y4098* **(B)** mRNA produced by the WT, Δ3′ UTR, Δ*hfq*, and Δ3′ UTR Δ*hfq* strains. Student’s *t*-test was used to calculate *p*-values. ^∗^*p* < 0.01. Means ± standard deviation (SD) from three independent experiments are shown.

Hfq commonly binds to 3′ ends of mRNA (Holmqvist et al., 2016) and regulates mRNA stability by protecting RNA from degradation (Zhang et al., 2003; Chao et al., 2012). To investigate whether Hfq is involved in the regulation of 3′ UTR-mediated *y1235* and *y4098* mRNA decay, we used an *hfq* single deletion strain (Zhu et al., 2016), and constructed an *hfq* and *y1235* or *y4098* 3′ UTR double deletion strain, and analyzed *y1235* and *y4098* mRNA levels in these strains by qRT-PCR. Deletion of *hfq* resulted in slightly decreased *y1235* and *y4098* mRNA levels relative to the WT strain (Figure 4). However, deletion of *hfq* in the 3′ UTR mutants caused a much stronger decrease in *y1235* and *y4098* mRNA levels relative to those in the 3′ UTR mutants (Figure 4), suggesting that Hfq protects their mRNA stability in a 3′UTR-dependent manner. In addition, *y1235* and *y4098* mRNA levels were much higher in the Δ*hfq* Δ3′ UTR strain than in the Δ*hfq* strains (Figure 4), suggesting that 3′ UTRs regulate *y1235’* and *y4098’* mRNA decay in an Hfq-independent manner.

### The Roles of RNases in the Expression of *y1235* and *y4098*

**FIGURE 5:**
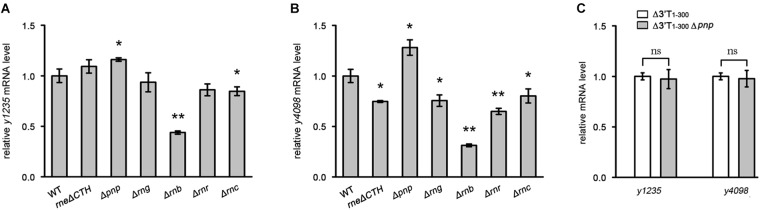
The effects of the major RNases on 3′ UTR-mediated mRNA turnover. The major RNases coding genes, CTH of *rne* [the carboxyl terminal half (CTH) region of RNase E], *pnp* (PNPase), *rng* (RNase G), *rnb* (RNase II), *rnr* (RNase R), and *rnc* (RNase III) were deleted, respectively. **(A,B)** Relative *y1235* **(A)** and *y4098* **(B)** mRNA levels in *Y. pestis* RNases mutants. **(C)** Relative *y1235* and *y4098* mRNA levels in *Y. pestis* Δ3′ UTR (white bars) and PNPase-Δ3′ UTR (gray bars) isogenic strains. Student’s *t*-test was used to calculate *p*-values. ^∗^*p* < 0.05, ^∗∗^*p* < 0.001. Means ± SD from three independent experiments are indicated.

3′ UTRs may be targeted by RNases to initiate mRNA decay (Maeda and Wachi, 2012; Lopez-Garrido et al., 2014). To investigate which RNases contribute to 3′ UTR-mediated mRNA decay, we analyzed *y1235* and *y4098* mRNA abundance in cells in which RNase E, RNase G, RNase II, RNase R, RNase III, or PNPase was mutated (Zhu et al., 2016). RNase E is essential, but deletion of its C-terminal CTH region impairs RNase E-mediated RNA degradation without causing cell death (Bouvier and Carpousis, 2011; Ow et al., 2000). Thus, we used a *Y. pestis* C-terminal truncated RNase E mutant (Zhu et al., 2016) to determine whether it is involved in 3′ UTR-mediated mRNA decay. Mutation of RNase E, RNase G, RNase II, RNase R, or RNase III did not increase the mRNA levels of *y1235* and *y4098*. However, mutation of PNPase caused increased *y1235* and *y4098* mRNA levels (Figures 5A,B), suggesting that PNPase might target these mRNAs for degradation. To determine whether the 3′ UTRs of *y1235* and *y4098* are targeted by PNPase, a PNPase mutation was generated in *Y. pestis* *y1235* 3′ UTR and *y4098* 3′ UTR deletion mutants. The levels of *y1235* and *y4098* mRNAs were similar in PNPase+ and PNPase- backgrounds (Figure 5C), indicating that PNPase is involved in 3′ UTR-mediated degradation of *y1235* and *y4098* mRNAs.

### The *y1235* and *y4098* 3′ UTRs Regulate Gene Expression in Response to Temperature Change

**FIGURE 6:**
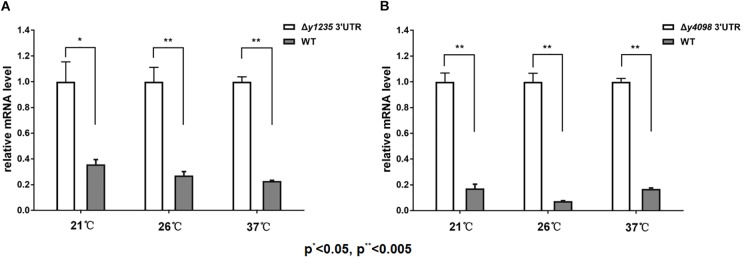
The effects of temperature on 3′ UTR-mediated mRNA turnover. qRT-PCR analysis of relative *y1235* **(A)** and *y4098* **(B)** mRNA levels in the WT and Δ3′ UTR mutant strain at 21°C, 26°C, and 37°C. Student’s *t*-test was used to calculate *p*-values. ^∗^*p* < 0.01, ^∗∗^*p* < 0.005. Means ± SD from three independent experiments are indicated.

The regulation of gene expression in a precise manner is critical for bacterial adaptation to changing environments. Recently, we reported that 3′ UTR-mediated *hmsT* mRNA decay in *Y. pestis* is affected by changes in temperature (Zhu et al., 2016). To test whether temperature change affects 3′ UTR-dependent stability of *y1235* and *y4098* mRNAs, we compared *y1235* and *y4098* mRNA levels in WT and 3′ UTR mutant strains at 21°C, 26°C, and 37°C. Figure 6A shows that the level of the *y1235* transcript was moderately high at 21°C (2.8-fold), but much higher at 26°C (3.8-fold) and 37°C (4.3-fold). To our surprise, the level of the *y4098* transcript was highest at 26°C (13.8-fold), although its level was also high at 21°C (5.8-fold) and 37°C (5.7-fold) (Figure 6B). The results suggest that the 3′ UTRs of *y1235* and *y4098* function as thermosensors and differentially regulate gene expression in response to temperature changes.

### The Effects of Ribosome Binding to 3′ UTRs

**FIGURE 7:**
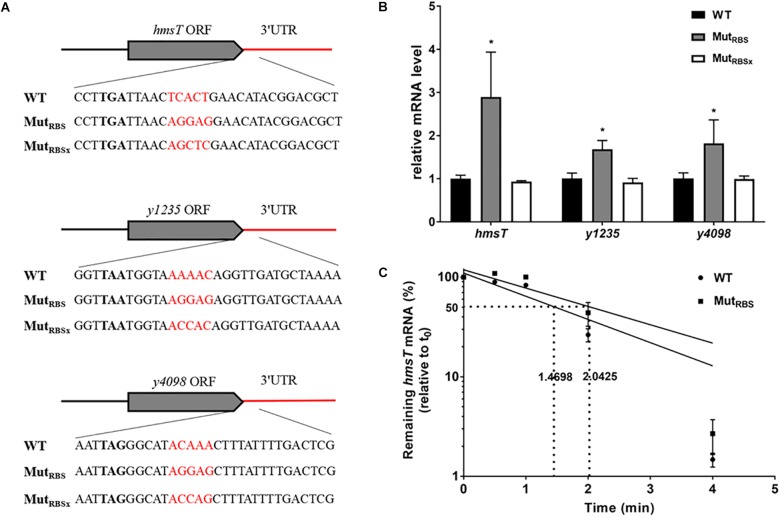
The effects of ribosome binding to 3′ UTR on mRNA decay. **(A)** Schematic representation of mutations introduced in 3′ UTRs of *hmsT*, *y1235*, and *y4098*. **(B)** qRT-PCR analysis of relative *hmsT*, *y1235*, and *y4098* mRNA levels in *Y. pestis* WT strain, in the strain with a 3′ UTR containing a ribosome binding site (RBS), and in the strain containing the 3′ UTR with a sequence not recognized by the ribosome (RBSx). Student’s *t*-test was used to calculate *p*-values. ^∗^*p* < 0.05. Means ± SD from three independent experiments are presented. **(C)** *hmsT* mRNA half-lives in the WT and 3′ UTR with RBS strains. Cells were grown at 26°C to an OD_600_ of ∼0.8, and rifampicin was added at time 0 to block RNA transcription. Samples were removed at time 0, 0.5 min, 1 min, 2 min, and 4 min. The percentage of mRNA remaining at each time-point was measured by qRT-PCR and compared with that at time 0. Dashed lines indicate the time at which half of the detected mRNA remained. The half-life of mRNAs was determined by linear regression analysis and is shown on the dashed lines. Error bars indicate the SD of the mean.

In general, 5′ UTRs are targeted by RNases to induce mRNA decay, while ribosome binding to a strong Shine-Dalgarno-like sequence (ribosome binding site, RBS) promotes mRNA stability (Agaisse and Lereclus, 1996; Sharp and Bechhofer, 2003; Daou-Chabo et al., 2009). Since 3′ UTRs may also be attacked by RNases to initiate mRNA decay, we wondered whether protein binding to 3′ UTR could affect mRNA stability. To answer this question, we introduced an RBS region into 5′ end of the 3′ UTR of *hmsT*, *y1235*, and *y4098* genes (Figure 7A). Introduction of an RBS region in the 3′ UTR resulted in a significant increase in the mRNA levels of all three genes (Figure 7B). However, introduction of a sequence (RBSx) not recognized by ribosomes did not affect mRNA levels (Figures 7A,B). To determine whether introduction of an RBS in the 3′ UTR affects post-transcriptional gene expression, the mRNA half-life of *hmsT* was measured by qRT-PCR. Consistent with the above results, introduction of an RBS region but not a non-specific sequence into the 3′ UTR increased the *hmsT* mRNA half-life (Figure 7C). Taken together, these results suggest that binding of some proteins, such as ribosomes, to the 3′ UTR can protect it from degradation, which in turn promotes the stability of upstream mRNA.

## Discussion

Degradation of mRNA is dependent on a rate-determining initial step in bacteria (Kushner, 2002; Bechhofer, 2009). mRNA is initially degraded by endonucleolytic enzymes. After endonucleolytic cleavage, exoribonucleases degrade the mRNA fragments, but ribosomes binding prevent initial mRNA endonucleolytic degradation of mRNA (Deneke et al., 2013). Thus, ribonucleases tend to degrade more readily the untranslatable regions of mRNA. Although 3′ UTRs are generally more stable than 5′ UTRs, subgenic-resolution oligonucleotide microarrays analysis of the positional patterns of transcript degradation *in E. coli* showed that some mRNAs have unstable 3′ UTRs, indicating that 3′ UTRs might also initiate mRNA decay (Selinger et al., 2003). 3′ UTRs have recently been shown to affect gene expression in bacteria by altering mRNA stability and translation in *trans* and *cis*, and thus have been classified as new post-transcriptional regulatory elements (Novick et al., 1993; Balaban and Novick, 1995; Felden et al., 2011; Ruiz de los Mozos et al., 2013; Laalami et al., 2014). Although a number of functional 3′ UTRs and their regulatory functions have been identified in bacteria, further work will be required to identify additional functional 3′ UTRs and their regulatory mechanisms.

We identified additional functional 3′ UTRs by focusing on long 3′ UTRs and found that among 3′ UTRs, those with long AU-rich regions are probably more important for mRNA expression regulation. Interestingly, mRNA levels were higher when six out of seven potential AU-rich 3′ UTRs were deleted, whereas mRNA levels were only slightly affected in the absence of 3′ UTRs with normal AU content (Figure 1). Together with the results of GFP reporter assays showing that these 3′ UTRs regulate heterologous gene expression (Figure 2), these results suggest that AU-rich long 3′ UTRs play a prominent role in regulating gene expression.

Why are AU-rich 3′ UTRs more likely to be involved in the regulation of mRNA decay? There are at least three possible reasons: (1) AU-rich sequences are preferentially cleaved by endoribonucleases such as RNase E and G; (2) AU-rich sequences do not tend to form stable secondary structures and thus can be readily degraded by exoribonucleases; (3) the structures formed by AU-rich sequences are more likely to undergo secondary structural changes in response to temperature change, which would facilitate the fine-tuning of mRNA degradation to changes in temperature. In addition, 3′ UTRs may contain other elements that regulate gene expression. For example, *icaR* expression is controlled by base pair interactions between 5′ and 3′ UTRs in *Staphylococcus aureus* (Ruiz de los Mozos et al., 2013). The 5′/3′-UTR interaction controls ribonuclease activity to regulate *hbs* mRNA stability in *Bacillus subtilis* (Braun et al., 2017). In addition, RNA binding proteins such as Hfq prefer to bind to AU-rich sequences and regulate mRNA stability (Zhang et al., 2003; Chao et al., 2012; Holmqvist et al., 2016). Taken together, it appears that mRNA turnover is regulated by different types of 3′ UTRs in bacteria.

Similar to *hmsT* and *slrA* (Liu et al., 2016; Zhu et al., 2016), *y1235* and *y4098*, for which PNPase is involved in 3′ UTR-mediated mRNA decay, contain Rho-dependent terminators. PNPase cannot degrade strong stem-loop structures and its function relies on the presence of PNPase-susceptible 3′ ends (Liu et al., 2016). Rho-independent terminators but not Rho-dependent terminators usually form a strong stem-loop structure on the end of 3′ UTRs. Thus, PNPase is more likely to cleave from the 3′ ends of mRNAs with Rho-dependent terminators. This could explain why PNPase plays an important role in mRNA decay when 3′ UTRs contain Rho-dependent terminators. However, *y1235* and *y4098* mRNA levels were increased by only 1.2-fold and 1.3-fold in the absence of PNPase, respectively, suggesting that other ribonucleases may contribute to 3′ UTR-mediated mRNA decay. In addition, 3′ UTRs containing a Rho-independent terminator such as *y2025* and *lrhA* might also regulate mRNA decay (Figures 1, 2). More than 21 ribonucleases are known in *E. coli* and most have *Y. pestis* homologs (Mackie, 2013). The ribonucleases involved in 3′ UTR-mediated mRNA degradation remain to be identified.

SmAP2 binds to 3′ UTRs and regulates mRNA stability in the crenarchaeum *Sulfolobus solfataricus* (Martens et al., 2017). Our results showed that Hfq protects mRNA from degradation in a 3′ UTR-dependent manner (Figure 4) and ribosome binding to the 3′ UTR promotes mRNA stability in *Y. pestis* (Figure 7C), suggesting that other RNA-binding proteins in bacteria might also regulate gene expression by binding to 3′ UTRs. RNA-binding proteins might mask endoribonucleolytic sites in 3′ UTRs or protect mRNA from degradation from 3′ ends by exoribonucleases. Several different mechanisms of 3′ UTR-regulated gene expression have been reported, but others remain to be elucidated (Ren et al., 2017). Further work should be directed toward identifying new functional 3′ UTRs and novel 3′ UTR-dependent regulatory mechanisms. This will increase our knowledge of how bacteria use 3′ UTR-dependent mRNA degradation to regulate gene expression in response to environmental changes.

## Materials and Methods

### Strains and Plasmids

Strains and plasmids used in this study are listed in Supplementary Table S1. *Y. pestis* KIM6+ was the WT strain. *Y. pestis* with deletions in 3′ intergenic regions and *hfq* was constructed by inserting a kanamycin (kan) and chloramphenicol (cat) resistance cassette, respectively, using the Lambda red protocol as previously described (Datsenko and Wanner, 2000; Sun et al., 2008). The 3′ UTR mutants of *hmsT*, *y1235*, and *y4098* genes were generated using the CRISPR-Cas12a-assisted genome editing tool (Yan et al., 2017). Overlapping PCR was used to insert 300 bp 3′ T regions of selected genes immediately downstream of the pAcGFP1 *gfp* stop codon, resulting in plasmids pAcGFP1-3′ T_1-300_. Plasmids encoding *gfp* fusion with 3′ UTR of *y1235* or *y4098* were constructed by reverse PCR using the corresponding pAcGFP1-3′ T_1-300_ as template. Strains and plasmids were constructed using the oligonucleotides listed in Supplementary Table S2. Cells were grown at 26°C in LB medium unless otherwise specified.

### Quantitative RT-PCR

qRT-PCR was performed as described previously with minor modifications (Sun et al., 2011). Briefly, cells in LB broth were grown overnight at 26°C with shaking at 250 rpm, diluted to an OD_600_ of ∼0.02, and then grown at 26°C in LB broth to an OD_600_ of ∼0.8. The RNeasy mini kit (Qiagen) was used to isolate total RNA from cells and DNase I (Invitrogen) was used to remove residual DNA. The purified RNA was used for quantitative PCR using the TaqMan^®^ RNA -to-C_T_^TM^ *1-Step* Kit (ABI) and CFX96TM Real-Time System (Bio-Rad) according to the user guide. mRNA quantity was normalized with respect to the 16S rRNA gene, and ratios of normalized mRNA quantities in different strains to the normalized quantities in the WT strain were calculated. The data presented represents the results of three independent experiments performed in triplicate. Probes and primer sets are listed in Supplementary Table S2.

### mRNA Half-Life Determination

mRNA half-life was determined as described previously with minor modifications (Bellows et al., 2012). WT *Y. pestis* (KIM6+) and 3′ UTR mutant strains in LB broth were grown overnight at 26°C with shaking at 250 rpm, diluted to an OD_600_ of ∼0.02, and then grown at 26°C to an OD_600_ of approximately ∼0.8. Rifampicin was added at a final concentration of 250 μg/ml to prevent initiation of transcription. Samples were collected at times 0, 0.5 min, 1 min, 2 min, and 4 min. RNA was extracted and analyzed by qRT-PCR, and the mRNA half-life was determined by linear regression analysis.

### 3′ RACE

3′ RACE was used to determine the transcription termination sites of *y1235* and *y4098* genes, as described previously (Zhu et al., 2016), using the 3′ RACE System for Rapid Amplification of cDNA Ends Kit (Invitrogen) according to the manufacturer’s instructions. RT-PCR products were separated using agarose gel electrophoresis, gel-purified, cloned into the pGEM-T vector, and transformed into *E. coli* cells. Transformants were picked for DNA sequencing and analysis.

### GFP Assay

Green fluorescent protein reporter analysis was performed as described previously (Zhu et al., 2016). Briefly, overnight cultures of *E. coli* strains harboring GFP reporters were diluted 1:1000 in triplicate and grown in 5 ml LB at 37°C to an OD_600_ of ∼0.35. Aliquots (100 μl) were then placed in 96-well Opti-Plates and GFP fluorescence was detected using a Multi label Reader (PerkinElmer) with an excitation wave length of 475 nm and an emission wavelength of 505 nm. The fluorescence of a control strain not expressing GFP was subtracted from the fluorescence of the samples, and relative fluorescence intensities were normalized against cell density (OD_600_ value).

## Author Contributions

Experiments were designed and manuscript was written by J-PZ, HZ, and Y-CS. Experiments were carried out by J-PZ, HZ, and X-PG. Manuscript review and modification were performed by J-PZ, HZ, X-PG, and Y-CS.

## Conflict of Interest Statement

The authors declare that the research was conducted in the absence of any commercial or financial relationships that could be construed as a potential conflict of interest.

## Supplementary Material

Figure S1 Mapping of the transcription termination site of *y1235* **(A)** and *y4098* **(B)** mRNA by 3′ rapid amplification of cDNA ends (RACE). 3′ RACE products were analyzed by agarose gel electrophoresis. Fragments of upper bands (red arrows) obtained by PCR amplification were purified, cloned into pUC19, and transformed into *E. coli* cells. The resulting clones were picked for sequencing. The corresponding regions of 3′ UTRs are underlined, and the specific primers used for 3′ RACE are labeled.

Table S1 Strains and plasmids employed in this study.

Table S2 Primers and probes used in this work.
