# Supplementary figures and images for: AU-Rich Long 3′ Untranslated Region Regulates Gene Expression in Bacteria
Source: Front Microbiol. 2018 Dec 12;9:3080. doi: 10.3389/fmicb.2018.03080 (PMC6299119; doi:10.3389/fmicb.2018.03080)

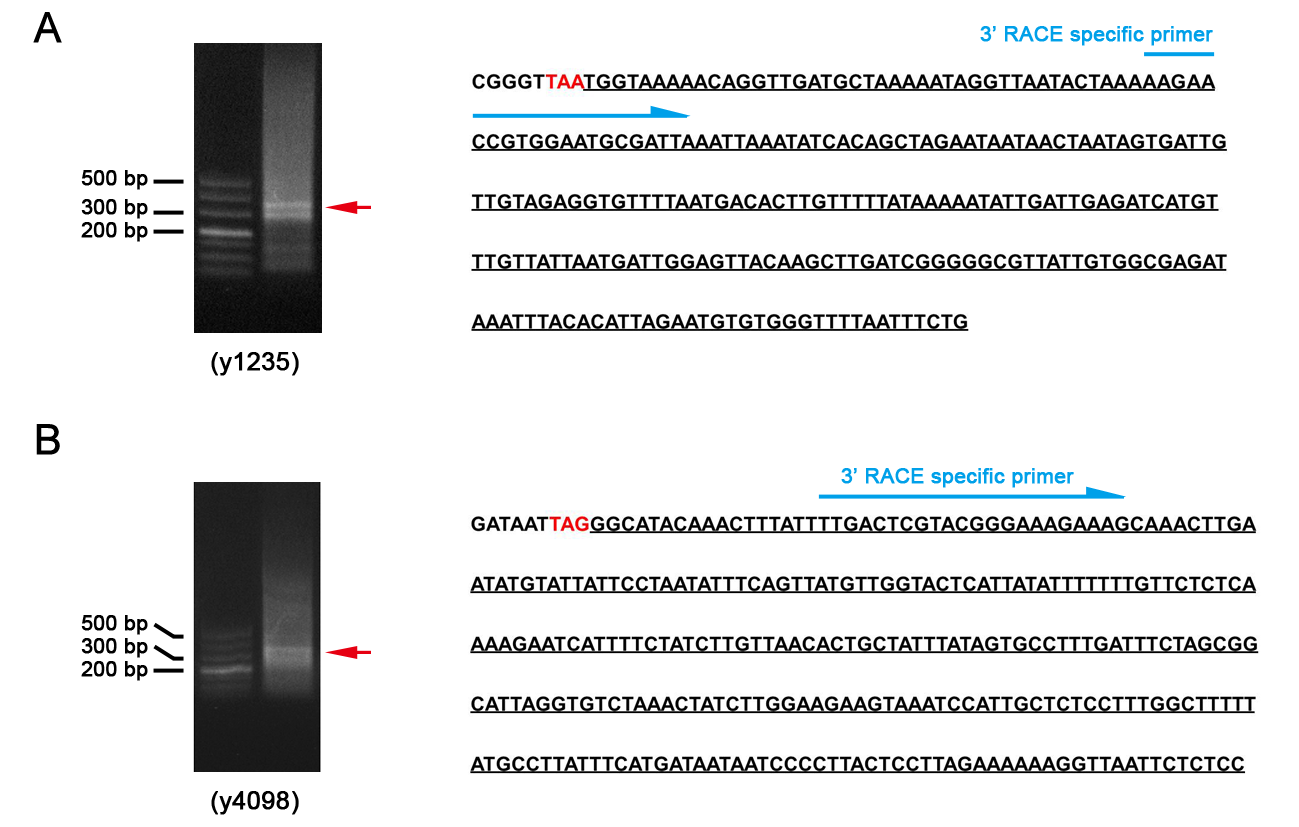

Supplement: Figure S1 — Mapping of the transcription termination site of y1235 (A) and y4098 (B) mRNA by 3′ rapid amplification of cDNA ends (RACE). 3′ RACE products were analyzed by agarose gel electrophoresis. Fragments of upper bands (red arrows) obtained by PCR amplification were purified, cloned into pUC19, and transformed into E. coli cells. The resulting clones were picked for sequencing. The corresponding regions of 3′ UTRs are underlined, and the specific primers used for 3′ RACE are labeled. [file Image_1.TIF]
