# Supplementary material for: AU-Rich Long 3′ Untranslated Region Regulates Gene Expression in Bacteria
Source: Front Microbiol. 2018 Dec 12;9:3080. doi: 10.3389/fmicb.2018.03080 (PMC6299119; doi:10.3389/fmicb.2018.03080)
Supplement: Table S1 — Strains and plasmids employed in this study. [file Table_1.DOCX]

**Supplementary Table S1. Strains and plasmids employed in this study**

| Names | Relevant characteristic(s) | Source |
| --- | --- | --- |
| Strains  Kim6+  *dsbA* Δ3’T_1-300_  *y0624* Δ3’T_1-300_  *y2025* Δ3’T_1-300_  *y1235* Δ3’T_1-300_  *lrhA* Δ3’T_1-300_  *y4098* Δ3’T_1-300_  *y0961* Δ3’T_1-300_  *cafA* Δ3’T_1-300_  *y2237* Δ3’T_1-300_  *y2419* Δ3’T_1-300_  *y3757* Δ3’T_1-300_  *hmsP* Δ3’T_1-300_  *amn* Δ3’T_1-300_  *y1288* Δ3’T_1-300_  *nadB* Δ3’T_1-300_  *y1235* Δ3’UTR  *y4098* Δ3’UTR  Δ*hfq*  *y1235* Δ3’UTR Δ*hfq*  *y4098* Δ3’UTR Δ*hfq*  *rne*ΔCTH  Δ*pnp*  Δ*rng*  Δ*rmb*  Δ*rnr*  Δ*rnc*  *y1235* Δ3’T_1-300_ Δ*pnp*  *y4098* Δ3’T_1-300_ Δ*pnp*  *hmsT* 3’UTR Mut_RBS_  *hmsT* 3’UTR Mut_RBSx_  *y1235* 3’UTR Mut_RBS_  *y1235* 3’UTR Mut_RBSx_  *y4098* 3’UTR Mut_RBS_  *y4098* 3’UTR Mut_RBSx_  Plasmids  pKD46-Cpf1-Amp    pAC-crRNA-Cm    pcrRNA-*hmsT*  pcrRNA-*y1235*  pcrRNA-*y4098*  pKD46  pKD3  pKD4  GFP-*rrnB*-TT  GFP-*y0264*-3’T_1-300_  GFP-*y2025*-3’T_1-300_  GFP-*lrhA*-3’T_1-300_  GFP-*y1235*-3’T_1-300_  GFP-*y4098*-3’T_1-300_  GFP-*y0961*-3’T_1-300_  GFP-*y2237*-3’T_1-300_  GFP-*nadB*-3’T_1-300_  GFP-*y1235*-3’UTR  GFP-*y4098*-3’UTR | Wild type (pCD1-)  *dsbA* 3’T_1-300_ deletion  *y0624* 3’T_1-300_ deletion  *y2025* 3’T_1-300_ deletion  *y1235* 3’T_1-300_ deletion  *lrhA* 3’T_1-300_ deletion  *y4098* 3’T_1-300_ deletion  *y0961* 3’T_1-300_ deletion  *cafA* 3’T_1-300_ deletion  *y2237* 3’T_1-300_ deletion  *y2419* 3’T_1-300_ deletion  *y3757* 3’T_1-300_ deletion  *hmsP* 3’T_1-300_ deletion  *amn* 3’T_1-300_ deletion  *y1288* 3’T_1-300_ deletion  *nadB* 3’T_1-300_ deletion  *y1235* 3’UTR deletion  *y4098* 3’UTR deletion  *hfq* deletion  *y1235* 3’UTR and *hfq* deletion  *y4098* 3’UTR and *hfq* deletion  *rne*CTH deletion  *pnp* deletion  *rng* deletion  *rmb* deletion  *rnr* deletion  *rnc* deletion  *y1235* 3’T_1-300_ and *pnp* deletion  *y4098* 3’T_1-300_ and *pnp* deletion  Introduction of RBS region in *hmsT* 3’UTR  Introduction of RBS similar sequence in *hmsT* 3’UTR  Introduction of RBS region in *y1235* 3’UTR  Introduction of RBS similar sequence in *y1235* 3’UTR  Introduction of RBS region in *y4098* 3’UTR  Introduction of RBS similar sequence in *y4098* 3’UTR  expressing λ Red recombinase and the nuclease Cas12a  CrRNA expression vector  Protospacer of *hmsT* in pAc-crRNA-Cm  Protospacer of *y1235* in pAc-crRNA-Cm  Protospacer of *y4098* in pAc-crRNA-Cm  *repA*101(ts) *bla* *araC* *P_araR_-Red*  Template plasmids for *hfq* deletion  Template plasmids for 3’ T_1-300_ deletion  *gfp::rrnB*-TT in pAcGFP1  *gfp:: y0264*-3’T_1-300_ in pAcGFP1  *gfp:: y2025*-3’T_1-300_ in pAcGFP1  *gfp:: lrhA*-3’T_1-300_ in pAcGFP1  *gfp:: y1235*-3’T_1-300_ in pAcGFP1  *gfp:: y4098*-3’T_1-300_ in pAcGFP1  *gfp:: y0961*-3’T_1-300_ in pAcGFP1  *gfp:: y2237*-3’T_1-300_ in pAcGFP1  *gfp:: nadB*-3’T_1-300_ in pAcGFP1  *gfp:: y1235*-3’UTR in pAcGFP1  *gfp:: y4098*-3’UTR in pAcGFP1 | Fetherston, 1992([1](#_ENREF_1))  This study  This study  This study  This study  This study  This study  This study  This study  This study  This study  This study  This study  This study  This study  This study  This study  This study  Zhu, 2016(2)  This study  This study  Zhu, 2016(2)  Zhu, 2016(2)  Zhu, 2016(2)  Zhu, 2016(2)  Zhu, 2016(2)  Zhu, 2016(2)  This study  This study  This study  This study  This study  This study  This study  This study  Yan, 2017(3)  Yan, 2017(3)  This study  This study  This study  Datsenko, 2000(4)  Datsenko, 2000(4)  Datsenko, 2000(4)  Zhu, 2016(2)  This study  This study  This study  This study  This study  This study  This study  This study  This study  This study |

**Reference**

1. Fetherston JD, Schuetze P, & Perry RD (1992) Loss of the pigmentation phenotype in Yersinia pestis is due to the spontaneous deletion of 102 kb of chromosomal DNA which is flanked by a repetitive element. *Molecular microbiology* 6(18):2693-2704.

2. Zhu H, Mao XJ, Guo XP, & Sun YC (2016) The hmsT 3' untranslated region mediates c-di-GMP metabolism and biofilm formation in Yersinia pestis. *Molecular microbiology* 99(6):1167-1178.

3. Yan MY*, et al.* (2017) CRISPR-Cas12a-Assisted Recombineering in Bacteria. *Applied and environmental microbiology* 83(17).

4. Datsenko KA & Wanner BL (2000) One-step inactivation of chromosomal genes in Escherichia coli K-12 using PCR products. *Proceedings of the National Academy of Sciences of the United States of America* 97(12):6640-6645.
