# Supplementary material for: AU-Rich Long 3′ Untranslated Region Regulates Gene Expression in Bacteria
Source: Front Microbiol. 2018 Dec 12;9:3080. doi: 10.3389/fmicb.2018.03080 (PMC6299119; doi:10.3389/fmicb.2018.03080)
Supplement: Table S2 — Primers and probes used in this work. [file Table_2.DOCX]

**Supplementary** **Table S2. Primers and probes used in this work**

| Primer name | Sequence 5’–3’ | Purpose |
| --- | --- | --- |
| *dsbA* Δ3’T_1-300_-F  *dsbA* Δ3’T_1-300_-R  *y0624* Δ3’T_1-300_-F  *y0624* Δ3’T_1-300_-R  *y2025* Δ3’T_1-300_-F  *y2025* Δ3’T_1-300_-R  *y1235* Δ3’T_1-300_-F  *y1235* Δ3’T_1-300_-R  *lrhA* Δ3’T_1-300_-F  *lrhA* Δ3’T_1-300_-R  *y4098* Δ3’T_1-300_-F  *y4098* Δ3’T_1-300_-R  *y0961* Δ3’T_1-300_-F  *y0961* Δ3’T_1-300_-R  *cafA* Δ3’T_1-300_-F  *cafA* Δ3’T_1-300_-R  *y2237* Δ3’T_1-300_-F  *y2237* Δ3’T_1-300_-R  *y2419* Δ3’T_1-300_-F  *y2419* Δ3’T_1-300_-R  *y3757* Δ3’T_1-300_-F  *y3757* Δ3’T_1-300_-R  *hmsP* Δ3’T_1-300_-F  *hmsP* Δ3’T_1-300_-R  *amn* Δ3’T_1-300_-F  *amn* Δ3’T_1-300_-R  *y1288* Δ3’T_1-300_-F  *y1288* Δ3’T_1-300_-R  *nadB* Δ3’T_1-300_-F  *nadB* Δ3’T_1-300_-R  *y1235* Δ3’UTR-F  *y1235* Δ3’UTR-R  *y4098* Δ3’UTR-F  *y4098* Δ3’UTR-R  Δ*hfq*-F  Δ*hfq*-F  *hmsT* 3’UTR mutation  crRNA top  *hmsT* 3’UTR mutation crRNA bottom  *hmsT* 3’UTR Mut_RBS_  oligo  *hmsT* 3’UTR Mut_RBSx_ oligo  *y1235* 3’UTR mutation crRNA top  *y1235* 3’UTR mutation crRNA bottom  *y1235* 3’UTR Mut_RBS_  oligo  *y1235* 3’UTR Mut_RBSx_ oligo  *y4098* 3’UTR mutation crRNA top  *y4098* 3’UTR mutation crRNA bottom  *y4098* 3’UTR Mut_RBS_  oligo  *y4098* 3’UTR Mut_RBSx_ oligo  GFP-3F  GFP-*y0624*r-fusion-F  GFP-*y0624*r-fusion-R  *y0624*r-R  GFP-*y2025*r-fusion-F  GFP-*y2025*r-fusion-R  *y2025*r-R  GFP-*lrhA*r*-*fusion-F  GFP-*lrhA*r-fusion-R  *lrhA*r-R  GFP-*y1235*r-fusion-F  GFP-*y1235*r-fusion-R  *y1235*r-R  GFP-*y4098*r-fusion-F  GFP-*y4098*r-fusion-R  *y4098*r-R  GFP-*y2237*r-fusion-F  GFP-y2237r-fusion-R  *y2237*r-R  GFP-*nadB*r-fusion-F  GFP-*nadB*r-fusion-R  *nadB*r-R  GFP-*y1235*-3'UTR-F  GFP-*y4098*-3'UTR-F  GFP-*y1235*-3'UTR-R  GFP-*y4098*-3'UTR-R  *hmsT*-TaqMan-F  *hmsT*-TaqMan-R  *dsbA*-TaqMan-F  *dsbA* -TaqMan-R  *y0624*-TaqMan-F  *y0624*-TaqMan-R  *y2025*-TaqMan-F  *y2025*-TaqMan-R  *y1235*-TaqMan-F  *y1235*-TaqMan-R  *lrhA*-TaqMan-F  *lrhA* -TaqMan-R  *y4098*-TaqMan-F  *y4098*-TaqMan-R  *y0961*-TaqMan-F  *y0961*-TaqMan-R  *cafA*-TaqMan-F  *cafA* -TaqMan-R  *y2237*-TaqMan-F  *y2237*-TaqMan-R  *y2419*-TaqMan-F  *y2419*-TaqMan-R  *y3757*-TaqMan-F  *y3757*-TaqMan-R  *hmsP*-TaqMan-F  *hmsP*-TaqMan-R  *amn*-TaqMan-F  *amn*-TaqMan-R  *y1288*-TaqMan-F  *y1288*-TaqMan-R  *nadB*-TaqMan-F  *nadB*-TaqMan-R  16S-rRNA-TaqMan-F  16S-rRNA-TaqMan-R    Probes(FAM-TAMRA)  *hmsT*-TaqMan-P  *dsbA*-TaqMan-P  *y0624*-TaqMan-P  *y2025*-TaqMan-P  *y1235*-TaqMan-P  *lrhA*-TaqMan-P  *y4098*-TaqMan-P  *y0961*-TaqMan-P  *caPA*-TaqMan-P  *y2237*-TaqMan-P  *y2419*-TaqMan-P  *y3757*-TaqMan-P  *hmsP*-TaqMan-P  *amn*-TaqMan-P  *y1288*-TaqMan-P  *nadB*-TaqMan-P  16S-rRNA-TaqMan-P | aataaaagtatgtgtgaattatttctgttatggaatcatgcc  gatgtgtaggctggagctgcttcg  aaacagtatgcggatgttgttacattcctgctgactcaaaaa  taacatatgaatatccttag  gggtcgcgagttcgagtctcgtttcccgctccaaattttttcat  gtgtaggctggagctgcttcg  cggttgattcgtgccattgaaaagggtctaccacgggatgcc  tgacatatgaatatcctccttag  tttattgtcaccggtatcaaagctattttccccggattacttta  agtgtaggctggagctgcttcg  ccctcatatatgcagaacaaaaatccacta=cattaaatga  tagtcacatatgaatatcctaattag  ttggttaaaccccatttggggttagcaatggtaaaaacgggt  taagtgtaggctggagctgcttcg  agggaatcacccccgtaatcagcaatcaataaattcgcattc  catcatatgaatatcctccttag  cattaatattggattccgattatttgacaggtgatgaagatta  agtgtaggctggagctgcttcg  atcctgttcttccactcagtagtggacagtatttttttgatccag  catatgaatatcctccttag  cttataaggaaagtgataaagtgataaatatattttctaaag  ctgataattaggtgtaggctggagctgcttcg  attcagaacgggataataaactaatctggctaaaaaagtaa  aaaacatatgaatatcctccttag  ttgaagaaaatatactcccattgttggggtgatagtcatctctg  agtgtaggctggagctgcttcg  actatccaataaatccatctaccatttatattgtctaatattttc  catatgaatatcctccttag  cctgcaagtcagccttcggcatttgggcgcgaatatcgcgtaag  tgtaggctggagctgcttcg  gccactgtataaccaagaacagtttgacgtggtgatgatgtaa  catatgaatatcctccttag  caagcggcgctctgttaaccagcacgatgcgattcatttaatgt  gtaggctggagctgcttcg  tacaatccaggcccagcagagatacaaactattgtcactgact  agcatatgaatatcctccttag  tcaggaggaatgaaaagaggatgattgatacaaaatagattt  aaagtgtaggctggagctgcttcg  tggttgaacttcagcgcgacatcacccccggtctgtcatgacata  tgaatatcctccttag  ttctggtggtacctgcatcatcacattcaaacacttatgatcgtgt  aggctggagctgcttcg  ttcacattgccaatgatttttccagactggaccgttcagtaacata  tgaatatcctccttag  agaatttgaagcccagtatttcagcagcgctcaccacgtaagttaa ttaagtgtaggctggagctgcttcg  ggagcaattaaacccatacgcgatcatttaggctaatactctatttcatatgaa  tatcctccttag  ttcttatcgatactcacgctggcgataaacacccaagaatatagt  gtaggctggagctgcttcg  ctctcgtaaattacgtacctttaatgagccaccgttccgctaacat  atgaatatcctccttag  cctgagttttgccagctcatcagtgatttctatctaaactattaagt  gtaggctggagctgcttcg  tgatcaaatagaaaacccacagccaaccattctgcatccataac  atatgaatatcctccttag  ccctgatcaaatagaaaacccacagccaaccattctgcatccataa ttaagtgtaggctggagctgcttcg  gtctgaaggtatttactcacctgagttttgccagctcatcagtgatca  tatgaatatcctccttag  ttggttaaaccccatttggggttagcaatggtaaaaacgggttaa  gtgtaggctggagctgcttcg  gctagagttataatgaacgccaagtctataagacgccaataaatta  caggcatatgaatatcctccttag  cttataaggaaagtgataaagtgataaatatattttctaaagct  gataattaggtgtaggctggagctgcttcg  cgggataataaactaatctggctaaaaaagtaaaaaaccatgaa  atggcaagacatatgaatatcctccttag  ataggttcttagttaaaaacaacaagcaaataaggaaaatataga gtgtaggctggagctgcttcg  aatccgttgcttatgttctccatcatggtcgaccagcaatgggct catatgaatatcctccttag  tactcactgaacatacggacgctctagt  tagagcgtccgtatgttcagtgagtatc  atgtacagtcttccccttgattaacaggaggaacatacgatggctcta  tgccagttattatttttaa  ttaaaaataataactggcatagagcctacgtatgttcgagctgttaatc  aaggggaagactgtacat  tatggtaaaaacaggttgatgctaaagt  tttagcatcaacctgtttttaccatatc  aatggtaaaaacgggttaatggtaaggagaggttgatgatgaaaata  ggttaatactaaaaagaac  aatggtaaaaacgggttaatggtaaccacaggttgatgatcaaaata  ggttaatactaaaaagaac  tgggcatacaaactttattttgactcgt  gagtcaaaataaagtttgtatgcccatc  caagtttgctttctttcccgtacgacataaaataaagctcctatgccct  aattatcagctttagaa  caagtttgctttctttcccgtacgacttaaaataaagctggtatgcccta  attatcagctttagaa  ccaccatggtgagcaagggcgccga  gatgagctgtacaagtgaaatctttttattcagcatttttttg  aaatgctgaataaaaagatttcacttgtacagctcatccatgc  atttgcggccgctccacagctagagtgggtacga  gatgagctgtacaagtgaatcacaccattcagaattttcga  aattctgaatggtgtgattcacttgtacagctcatccatgc  atttgcggccgcctagtaacatctaaatagattcctaacag  gatgagctgtacaagtgagtagtctgtttttaattcatcatca  tgaattaaaaacagactactcacttgtacagctcatccatgcc  atttgcggccgcgacacgttttgagtgttatttcataaat  gatgagctgtacaagtgatggtaaaaacaggttgatgctaa  gcatcaacctgtttttaccatcacttgtacagctcatccatgc  atttgcggccgcgctagagttataatgaacgccaagt  gatgagctgtacaagtgaggcatacaaactttattttgactcg  caaaataaagtttgtatgcctcacttgtacagctcatccatgc  atttgcggccgcccatgaaatggcaagaggaga  gatgagctgtacaagtgactaattttgggccacaaaaaggc  ctttttgtggcccaaaattagtcacttgtacagctcatccatgc  atttgcggccgcccgcgcaataatggtgtatttaatc  gatgagctgtacaagtgacacattctgaatccataagattg  ttatggattcagaatgtgtcacttgtacagctcatccatgc  atttgcggccgcccaattagcgtgaaataagaatcgg  gcggccgcgactctagaattccaactgagcgccggt  gcggccgcgactctagaattccaactgagcgccggt  aattctagagtcgcggccgccagaaattaaaacccacacattctaatgtgta  aattctagagtcgcggccgctcttgccatttcatggttttttacttt  atcaggctctggtacggatttc  acgcaccacgatatctcttgaac  aactgtacagactcctggcgat  atgcagcgtcgtagtcttca  ccgctgtatctcttacctgacga  cataaatccggttacccatgagt  gttcgggagagtattggtgttgt  cagactatggtagagctgattgctc  cgatgctgattggttggcg  cgataattcatccggcctgc  gcccacgctttggtattgtt  catccatcaccactaaaggcact  ccttagtcactttatggtcagaaca  gctaagtatgatgttacaggcaataat  tgatgattgcattgcaggatga  ggatattggactagagggtaagaaga  tggagttcacccgcgaata  cgaaaataggttgcttcccagt  ttgctactttcaggttgtgtgac  gattaagatcttgcaacggtgtc  tcggaacgcttgaatctgtc  gataacctgcgcgttagcca  taccgaacggcttgaggtca  ccaatcaccgtcgtatcacg  catcatcaactgacgctaccg  attgcggttgtaattacgcac  cagggcaatacagcaccaca  tcaagcagcgtcatttgctc  gctgtggcagtgggctta  cccgtcaatctgcacctgt  gtgatatcgttgcccgagc  tggctgatatccagatacatgc  gcgaagaaccttacctactcttgac  gcacctgtctcacagttcccg  cacagcatcgcgaatggccg  cgtaatgtctttatcaaggcgggtatcagt  actggaaggcgcaatacctgaagagttc  cattggcactgcctttaatggctggac  atggcggttaatcctgattggctgca  tggattatcagttccagcccggagag  ctccttatgctaaccataatgtaattcatgg  tgtatagggatgatatgagccaagaggat  attccagaaatgacggacaagctggagc  ataccgcctgccattcagggaacgac  agagtgacagtccggcgtgcgttaagt  tcgtgacaatgtgacgctgaaaggtgag  cgcatcatcaggatgatgaactgggtgt  ctcgacccgcactgttcagggaatatct  cgccttaatgattgcccaacgttcatt  tcgaccatgaaatgaagcgcctcggt  cacagaatttggcagagatgctaaagtgcc | strain construction  strain construction  strain construction  strain construction  strain construction  strain construction  strain construction  strain construction  strain construction  strain construction  strain construction  strain construction  strain construction  strain construction  strain construction  strain construction  strain construction  strain construction  strain construction  strain construction  strain construction  strain construction  strain construction  strain construction  strain construction  strain construction  strain construction  strain construction  strain construction  strain construction  strain construction  strain construction  strain construction  strain construction  strain construction  strain construction  strain construction  strain construction  strain construction  strain construction  strain construction  strain construction  strain construction  strain construction  strain construction  strain construction  strain construction  strain construction  cloning  cloning  cloning  cloning  cloning  cloning  cloning  cloning  cloning  cloning  cloning  cloning  cloning  cloning  cloning  cloning  cloning  cloning  cloning  cloning  cloning  cloning  cloning  cloning  cloning  cloning  qRT-PCR  qRT-PCR  qRT-PCR  qRT-PCR  qRT-PCR  qRT-PCR  qRT-PCR  qRT-PCR  qRT-PCR  qRT-PCR  qRT-PCR  qRT-PCR  qRT-PCR  qRT-PCR  qRT-PCR  qRT-PCR  qRT-PCR  qRT-PCR  qRT-PCR  qRT-PCR  qRT-PCR  qRT-PCR  qRT-PCR  qRT-PCR  qRT-PCR  qRT-PCR  qRT-PCR  qRT-PCR  qRT-PCR  qRT-PCR  qRT-PCR  qRT-PCR  qRT-PCR  qRT-PCR  qRT-PCR  qRT-PCR  qRT-PCR  qRT-PCR  qRT-PCR  qRT-PCR  qRT-PCR  qRT-PCR  qRT-PCR  qRT-PCR  qRT-PCR  qRT-PCR  qRT-PCR  qRT-PCR  qRT-PCR  qRT-PCR  qRT-PCR |
